# Integrated approaches for increasing plant yield under salt stress

**DOI:** 10.3389/fpls.2023.1215343

**Published:** 2023-07-18

**Authors:** Irshad Ahmad, Guanglong Zhu, Guisheng Zhou, Muhammad Usama Younas, Mohamed Suliman Eltyeb Suliman, Jiao Liu, Yi ming Zhu, Ebtehal Gabralla Ibrahim Salih

**Affiliations:** ^1^ Joint International Research Laboratory of Agriculture and Agri-Product Safety of the Ministry of Education of China, Yangzhou University, Yangzhou, China; ^2^ Key Lab of Crop Genetics & Physiology of Jiangsu Province, Yangzhou University, Yangzhou, China; ^3^ Department of Crop Genetics and Breeding, College of Agriculture, Yangzhou University, Yangzhou, China; ^4^ Faculty of Forestry, University of Khartoum, Khartoum North, Sudan

**Keywords:** salt stress, morpho-physiological and biochemical activity, nutrient uptake, CRSIPER-Cas9, genes, yield

## Abstract

Salt stress affects large cultivated areas worldwide, thus causing remarkable reductions in plant growth and yield. To reduce the negative effects of salt stress on plant growth and yield, plant hormones, nutrient absorption, and utilization, as well as developing salt-tolerant varieties and enhancing their morpho-physiological activities, are some integrative approaches to coping with the increasing incidence of salt stress. Numerous studies have been conducted to investigate the critical impacts of these integrative approaches on plant growth and yield. However, a comprehensive review of these integrative approaches, that regulate plant growth and yield under salt stress, is still in its early stages. The review focused on the major issues of nutrient absorption and utilization by plants, as well as the development of salt tolerance varieties under salt stress. In addition, we explained the effects of these integrative approaches on the crop’s growth and yield, illustrated the roles that phytohormones play in improving morpho-physiological activities, and identified some relevant genes involve in these integrative approaches when the plant is subjected to salt stress. The current review demonstrated that HA with K enhance plant morpho-physiological activities and soil properties. In addition, *NRT* and *NPF* genes family enhance nutrients uptake, *NHX1*, *SOS1*, *TaNHX*, *AtNHX1*, *KDML*, *RD6*, and *SKC1*, maintain ion homeostasis and membrane integrity to cope with the adverse effects of salt stress, and *sd1/Rht1*, *AtNHX1*, *BnaMAX1s*, *ipal-1D*, and *sft* improve the plant growth and yield in different plants. The primary purpose of this investigation is to provide a comprehensive review of the performance of various strategies under salt stress, which might assist in further interpreting the mechanisms that plants use to regulate plant growth and yield under salt stress.

## Introduction

The world faces a tremendous challenge in crop production (López-Marqués et al., 2020). According to (FAO, 2017), the human population will increase to 10 billion, and the requirements for cereals and livestock production will exceed 60 percent (Springmann et al., 2018). Agriculture growth depends on productivity achieved through increased crop yields. However, the higher yield was only achieved during the green revolution period (López-Marqués et al., 2020). The percentage increase in yield has decreased after the green revolution.

Salt stress is one of the most important environmental factors, significantly decreasing crop growth and yield worldwide (Zörb et al., 2019; Negi et al., 2020). Salt stress affects approximately 20 percent of all agricultural lands and 33 percent of irrigated agricultural lands (FAO, 2017). The neutral salt concentrations of sodium chloride (NaCl) and sodium sulfate (Na_2_SO_4_) caused salt stress in soil (Van Zelm et al., 2020). The higher accumulation of NaCl in the soil depletes the water content and harms plants. It thus causes toxic effects from the sodium and chloride ions in plants (Ahmad et al., 2022b). To cope with the negative effects of salt stress’s, plants use various responses, such as regulating gene expression and stimulating hormones (Raza et al., 2022; Feng et al., 2023). During salt stress, different kinds of strategies can be used to increase plant growth and yields. Currently, researchers and growers realize the importance of identifying suitable cultivars (Jiang et al., 2022), nutrient absorption (Adil et al., 2022), the roles of hormones (Ahmad et al., 2022a), and gene identification (Tang et al., 2022) under salt stress (Adil et al., 2022; Jiang et al., 2022; Tang et al., 2022; Ahmad et al., 2022b).

Different studies have shown that the regulation of genes under salt stress is affected by numerous transcriptional cascades (Wu et al., 2019). Abscisic acid (ABA) and gibberellin (GA), both acting as endogenous signaling hormones and are essential regulators of salt stress (Pu et al., 2019; Ahmad et al., 2022b). *WRKY* genes in cotton respond to salt stress via ABA signaling and regulate the production of reactive oxygen species (ROS) in plant cells (Yan et al., 2014). In wheat, *MYB* genes respond to salt stress by regulating ion homeostasis in order to control osmotic pressure and lower ROS concentrations (Song et al., 2020). Understanding the molecular mechanism underlying salt resistance in plants is essential for improving crop quality and yield, and this can only be achieved by studying different salt-tolerant genes in plants (Wang et al., 2022).

Salt stress affects nutrient absorption and disturbs plant metabolic activities such as lipid and carbohydrate metabolism, which reduce crop growth and yield (Parida and Das, 2005; Zafar et al., 2022). In addition, salt stress affects the plant’s root system. It creates osmotic stress due to the elevated sodium (Na^+^), resulting in a water shortage in plant cells and thus affecting water potential (Ekinci et al., 2022). Due to the imbalances of nutrient availability in soil, salt stress thus causes ion toxicity in different plants (Ali et al., 2021).

**Figure 1:**
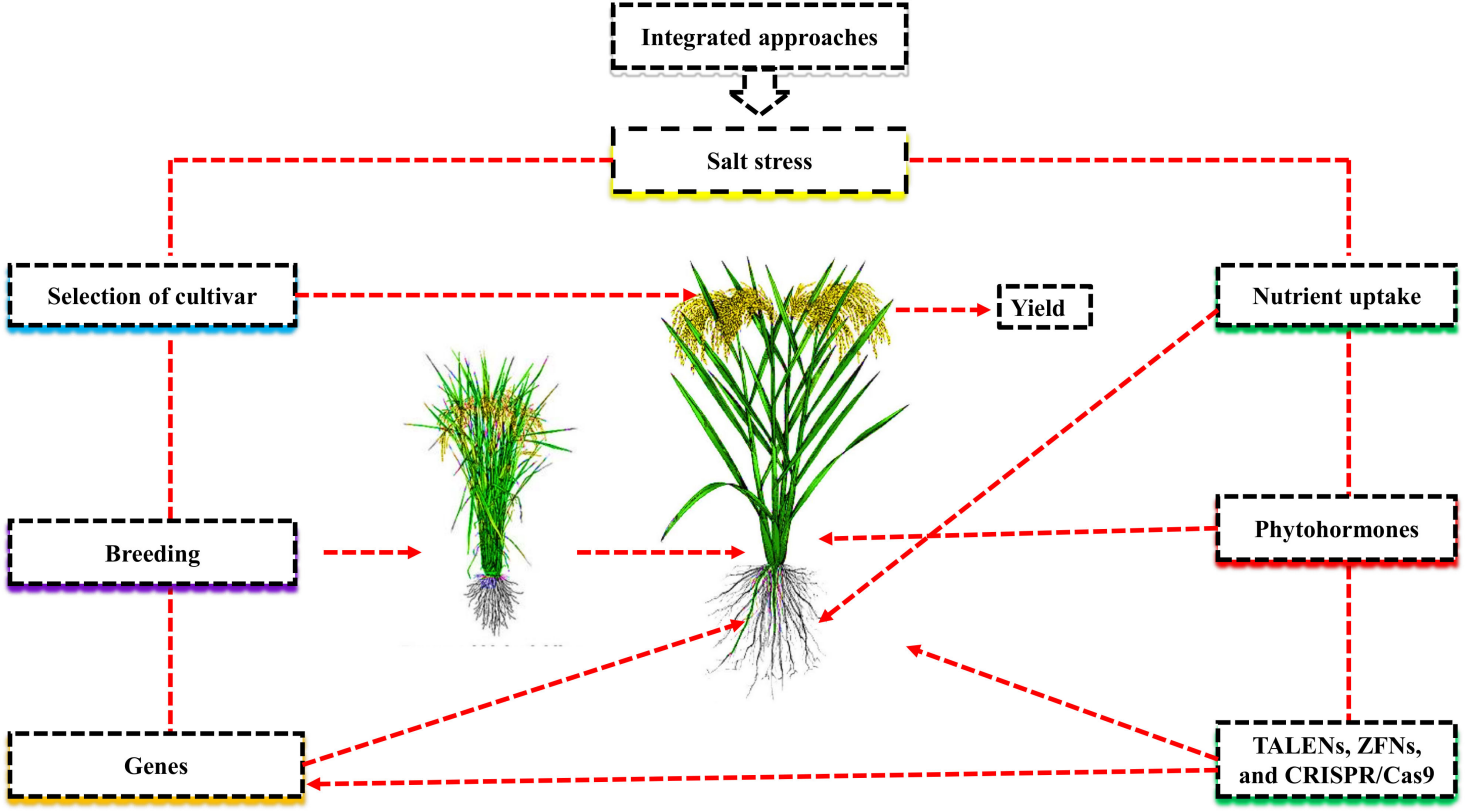
Integrated approach mitigate the negative effects of salt stress and improve plant growth and yield.

Introducing suitable cultivars and desirable genes, etc., has been widely investigated to improve crop growth and productivity under salt stress (Chattha et al., 2020). However, these approaches are time-consuming and costly. In order to improve the growth and yield of desirable cultivars, plant genomic editing with the CRISPR/Cas9 system is currently being used. Nevertheless, the regulation of genome-edited crops is still unknown, especially under abiotic stresses (Razzaq et al., 2019). Applying seed priming, nutrient management, and phyto-hormones to overcome the adverse effects of salt stress can suggest promising conclusions for various plants to improve yield (Ahmad et al., 2022a; Ahmad et al., 2022b; **Figure 1**). In this review, we examined how salt stress affects nutrient uptake and utilization in various crops under salt stress from the aspects of breeding salt-tolerant varieties, identifying salt-resistant genes, and using CRISPR-Cas9 tools for genome editing (**Table 1**).

**Table 1:** Different genes improve plant growth and yield under salt stress.

Plants	genes	Genes functions in plants	Salt stress	References
Cotton	*WRKY*,	Regulate ROS production in plant cell	Enhance salt tolerance via ABA signaling and regulate the production of ROS	(Yan et al., 2014)
Tomato	*MYB*	Prevent plant cell membrane from injury	Enhance salt tolerance via regulating of ROS production	(Cui et al., 2018)
Wheat	*MYB*	Regulates ion homeostasis	Enhance salt tolerance via regulating osmotic pressure and lower production of ROS	(Song et al., 2020)
*Arabidopsis*	*MYB*	Regulates antioxidant enzymes and cuticle formation	Enhance salt tolerance	(Zhang et al., 2020)
*Arabidopsis*	*NPF6.3*, *NRT1.1* or *CHL1*	NO3^-^	Enhance salt tolerance	(Liu et al., 1999)
Cotton	GA2ox7	Improve plant growth and development and biological process, enhance the content of ABA and IAA	Enhance salt tolerance and upregulated via GA	(Ahmad et al., 2022b)
*Arabidopsis thaliana*	*XERICO* and *GASAA*	Improve plant growth and development and biological process	Enhance salt tolerance and upregulated via GA	(Ahmad et al., 2022b)
Rice	*GA2ox, GA2ox5*, and *GA2ox6*	Improve plant growth and development and biological process, crop yield	Enhance salt tolerance and upregulated via GA	(Ahmad et al., 2022b)
Potato	*GA2ox*	Improve plant growth and development and biological process, crop yield	Enhance salt tolerance and upregulated via GA	(Ahmad et al., 2022b)
Tomato	*TaNHX*	Reduce the uptake of Na^+^	Enhance salt tolerance	(Liu et al., 2020)
Rice	*TaNHX*	Reduce the uptake of Na^+^	Enhance salt tolerance	(Liu et al., 2010)
Tomato	*AtNHX1*	Enhance K^+^ retention	Enhance salt tolerance	(He and He, 2023)
Rice	*KDML*, *RD6*, and *SKC1*	Improve seedling growth, maintenance of ionic homeostasis, and maintaining membrane integrity,	Enhance salt tolerance	(Nounjan et al., 2018; Pamuta et al., 2022)
Cereals	*sd1/Rht1*	Increase plant yield	Enhance salt tolerance	(Zheng et al., 2022)
wheat	*AtNHX1*	Increase plant yield	Enhance salt tolerance	(Sharma et al., 2022)
Brassica napus *L.*	BnaMAX1s (Dwarf gene)	Improve hormone biosynthetic activity and Increase plant yield	Enhance salt tolerance	(Zheng et al., 2020)
tomato	*sft*	improve photosynthetic activity, enhanced dry matter accumulation in the sink, and increased plant yield	Enhance salt tolerance	(Krieger et al., 2010)

## Different approaches for increasing plant growth and yield

### Effects of salt stress on crop growth and development

Salt stress is one of the major abiotic stresses that causes seed dormancy and reduces normal plant growth and yield. Soluble salt at higher concentrations causes both osmotic and ionic stresses in soil, which lead to secondary stresses such as nutritional imbalance and oxidative stress. In the root zone, the higher concentrations of salts cause higher osmotic pressure in the soil compared to plant cells, which reduces the capability of plants to uptake water and nutrients (El-Mageed et al., 2022). When plants are subjected to high salt stress, the soil solution becomes more hyper-osmotic and causes the root cells to lose water, resulting in plant senescence or wilting (Verma et al., 2022). Osmotic stress, caused by a lack of water in plant tissues, primarily reduces leaf growth and causes a reduction in shoots and reproductive growth. (Chourasia et al., 2022). Secondary stress, such as oxidative stress, mainly occurs due to the higher production of ROS, which contributes to the primary effects of salt stress described above (Kaur et al., 2023). In addition, the overproduction of ROS in plants increases the fluidity and permeability of cell membranes and degrades functional and structural proteins under salt stress (Sharma et al., 2023).

### Salt stress and genes involve in plant’s nutrient uptake

**Figure 2:**
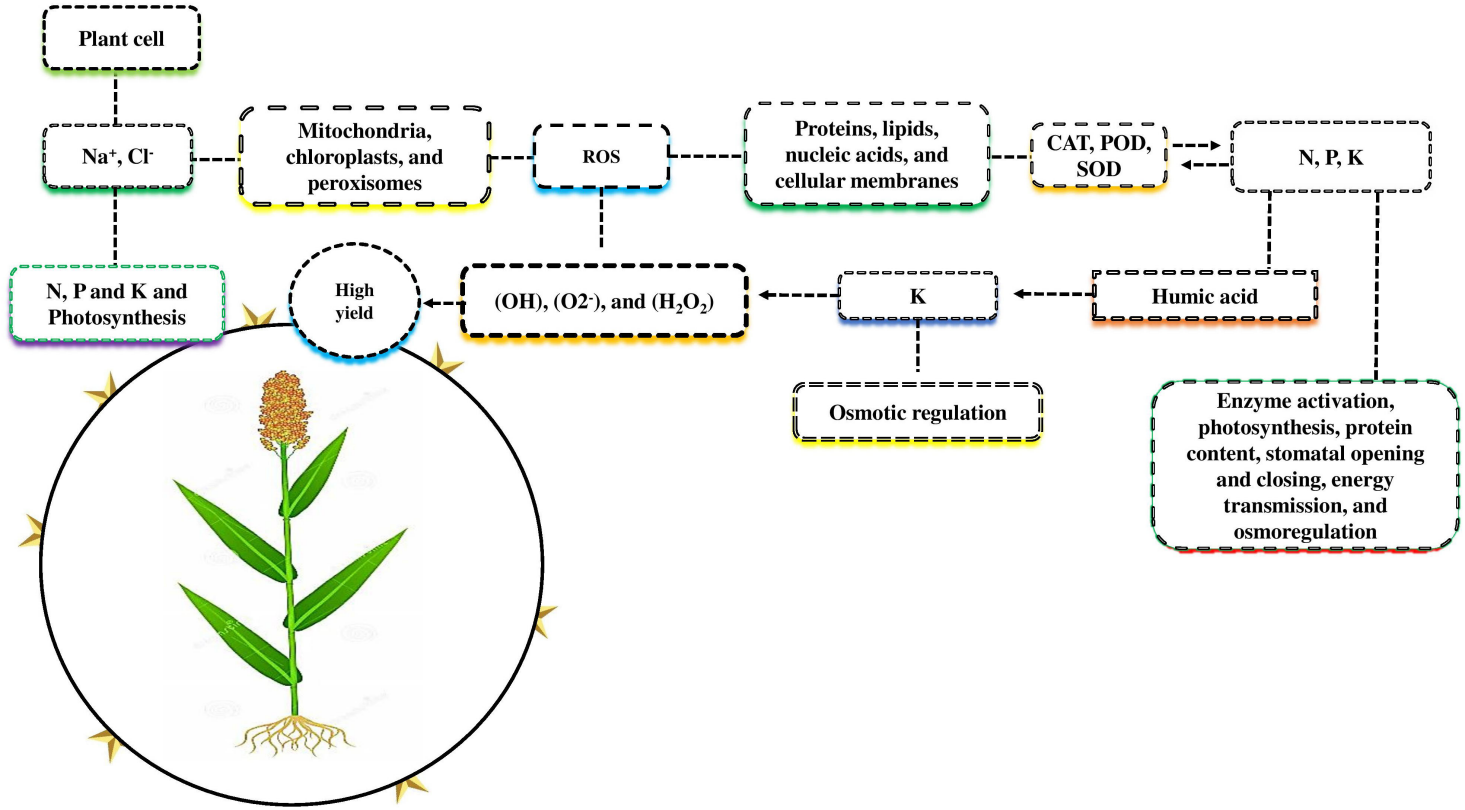
Nutrients mitigate the adverse effects of salt stress. Plant cells accumulate salt-effected ions such as Na^+^ and Cl^-^ that reduce N, P, and K uptake and photosynthetic activity. Plants organelles such as mitochondria, chloroplast, and peroxisome are exposed to higher salt stress, including the hydroxyl radical (OH), superoxide (O2^-^), and hydrogen peroxide (H_2_O_2_), produce more reactive oxygen species (ROS), which negatively affect protein, lipids nucleic acid, and cellular damage. For self-defense, plants produce different antioxidant enzymes, such as CAT, POD, and SOD, to eliminate the negative effects of salt stress. Macronutrients N, P and K play a major role in enzyme activation photosynthesis, protein content, stomatal opening and closing, energy transmission, and osmoregulation under salt stress. K play a vital role in osmotic regulation compared to N and P. The combined application of K with humic acid counters the negative effects of ROS and improves the morph-physiological activity and yield of various crops.

Due to salt-induced osmotic stress, plant growth and nutrient uptake decreased under salt stress. (Abbas et al., 2022). The uptake of nutrients by roots and the efficiency of photosynthesis by leaves are sources of plant development. Plant cells accumulate salt-affected soil ions (Na^+^, and Cl^-^) that inhibit nitrogen (N), phosphorus (P), and potassium (K) uptake and photosynthesis (Zhang et al., 2011; Ait-El-Mokhtar et al., 2020; **Figure 2**). Many plant organelles, including mitochondria, chloroplasts, and peroxisomes, produce more reactive oxygen species (ROS) when subjected to higher salt stress, such as hydroxyl radical (OH), superoxide (O2^-^), and hydrogen peroxide (H_2_O_2_). Proteins, lipids, nucleic acids, and cellular membranes were all negatively affected due to the higher accumulation of ROS in plants (Zulfiqar et al., 2019). Plants protect themselves against ROS by producing different antioxidant enzymes such as catalase, ascorbate peroxidase, and superoxide dismutase (Ahmad et al., 2022a).

Different mineral nutrients and organic amendments have been widely used to improve salt resistance and nutrient uptake in different kinds of crop species. Among these nutrients, N, P, and K are essential for plants as they are involved in different physiological and biochemical processes in plant growth and yield (Ahmad et al., 2022a). Previous studies demonstrated that macronutrients are required for various plant cellular processes, such as enzyme activation, photosynthesis, protein content, stomatal opening and closing, energy transmission, and osmoregulation under salt stress (Ashraf et al., 2013; Taha et al., 2020). K is suggested to be more efficient for osmotic regulation as compared to N and P under salt stress (Abbas et al., 2022). A key factor in plant salt resistance is the higher uptake of potassium over sodium (Zrig et al., 2021). Abbas et al. (2022) demonstrated that the application of K with humic acid (HA) enhanced different physiological and biochemical activities, such as nutrient uptake, water relations, stomatal conductance, and enzymes activation to counter the adverse effects of ROS (Abbas et al., 2022). HA, are organic compounds that are necessary for improving soil properties and plant growth (Ampong et al., 2022). The corresponding findings were supported by (Ali et al., 2019), who demonstrated that K with HA, increased salt resistance in sorghum by increasing its nutrient uptake and antioxidant activities, and reducing ROS production. The current review showed that K in combination with HA improved different morpho-physiological activities in plants. The application of HA with N and P or other molecules, such as fulvic acid (FA), which enhances soil qualities and plant growth in different crops under salt stress, is still not well understood.

**Figure 3:**
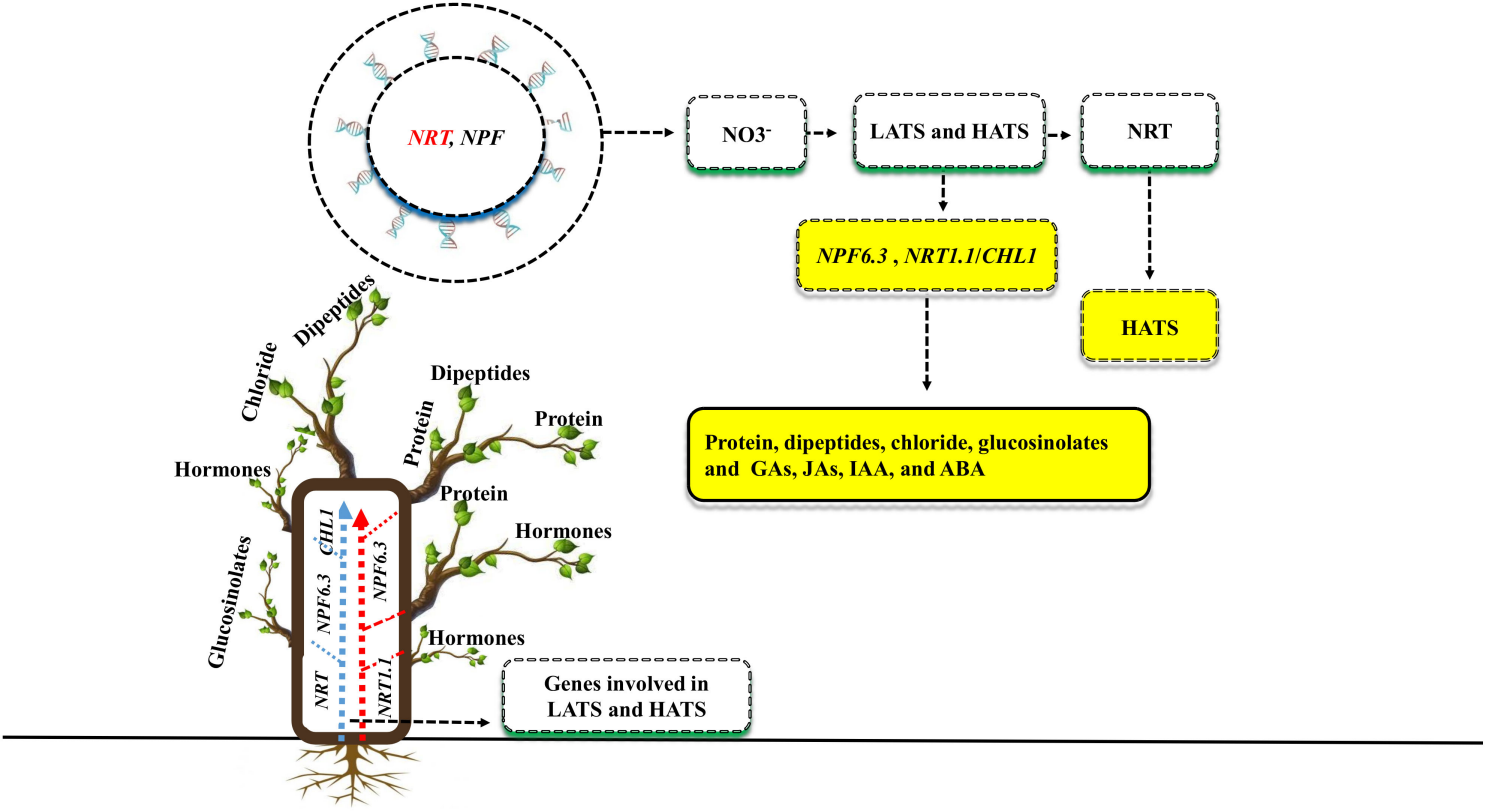
Different genes are involved in plant nutrient uptake and enhanced plant growth and yield under salt stress. Two gene families, *NRT* and *NPF*, enhance the uptake of NO3^-^ via two transportation methods: low-affinity transport systems (LATS) and high-affinity transport systems (HATS). *RNT* genes are especially involved in HATS, while *NPF* is involved in LATS. Some genes, such as *NPF6.3* and *NRT1.1/CHL1*, are involved in the dual-transport system. The genes *NPF6.3* transport a variety of substrates such as protein, dipeptides, chloride, glucosinolates, gibberellins (GAs), jasmonate (JAs), indole-3-acetic acid (IAA), abscisic acid (ABA) to the various parts of the plants.

Plant nutrient use efficiency and yield can be increased by identifying critical genes (Kumar et al., 2021). Both the *NRT* and *NPF* gene families have been recently discovered to be involved in nitrate uptake and transport throughout the plant (Fan et al., 2017; **Figure 3**). There are two types of transport systems involved in NO3^-^ uptake: low-affinity transport systems (LATS) and high-affinity transport systems (HATS) (Faure et al., 2021). Many *NRT* family genes have been identified as having high affinity, whereas *NPF* are thought to function as the primary components of the LATS for NO3^-^ at high concentrations (Li et al., 2021). Previous studies showed that some *RNT* and *NPF* family genes are involved in the dual-transport system; for example, in *Arabidopsis*, the *RNT* and *NPF* genes such as *NPF6.3*, *NRT1.1* or *CHL1*, was identified in both high- and low-affinity nitrate uptakes (Liu et al., 1999). The *NPF6.3* gene transport a variety of substrates, including protein concentration, dipeptides, chloride, glucosinolates, and plant hormones such as gibberellins (GAs), jasmonates (JAs), indole-3-acetic acid (IAA), and abscisic acid (ABA) (Fan et al., 2017; Chao et al., 2021). In *Arabidopsis*, the gene families *NRT* and *NPF* were crucial for nitrate uptake and transport to other parts of the plant. However, the remarkable performance of the *NPF6.3* gene under salt stress, as well as the identification of *NRT* and *NPF* low-to-high affinities genes in other plant species, remain unclear.

### Effects of salt stress on physiological and biochemical activities

Nevertheless, internally, plants have developed comprehensive resistance systems to cope with the adverse effects of ROS (Wang et al., 2023). SOD, CAT, POD, GR, and APX are ROS-scavenging and mediate in the reaction cycle of antioxidant chemicals, including ascorbic acid (AsA) and glutathione (GSH) (Apel and Hirt, 2004; Dietz et al., 2006; Türkan and Demiral, 2009).

Several studies demonstrated that salt stress reduces the physiological activities of wheat, barley, maize, sunflower, rice, tomatoes, and beets (Turan et al., 2009; Shahbaz et al., 2011; Shiyab et al., 2013; Umnajkitikorn et al., 2013; Zeeshan et al., 2020; He et al., 2022). In wheat and barley, the negative effects of salt stress may be due to a reduction in stomatal conductance or the excessive production of ROS in plants, which can enhance oxygen-induced cellular damage. Similarly, Netondo et al. (2004) in sorghum showed that changes in stomatal conductance and the concentration of cellular CO_2_ were positively correlated during salt stress, showing that stomatal conductance was a key factor that played an important role in plant net photosynthesis (Netondo et al., 2004). It has been confirmed that stomatal conductance plays a crucial role in net photosynthesis in wheat, barley, and sorghum, but the detailed mechanism in various species is still unknown.

### Genes improve plant physiological and biochemical activities under salt stress

Genes play an important role in reducing abiotic stresses in plants by facilitating their growth and development, nutrient uptake, and carrying them from one part of the plant to another. Salt-related genes, such as *SKC1*, *CDPK*, and MAPK pathways, overlay-sensitive (SOS) pathways, PAL and CHS, actively maintain response to salt stress in plants (Pamuta et al., 2022). *SOS1* and *NHX1* genes encode antiporter Na^+^/H^+^; however, *SOS1* is located on the plant plasma membrane (Du et al., 2023). The *SOS1* gene regulates the transport of Na^+^ from roots to the shoots of the plant (Liu et al., 2019). The *TaNHX* gene enhance plant tolerance against salt stress due to less uptake of Na^+^ and its translocation to the shoots in tomatoes and rice (Liu et al., 2010). The performance of the protein, *NHX* has been widely investigated in various crops such as tomatoes, rice, and cotton (Liu et al., 2010; Gouiaa and Khoudi, 2015). Though, in tomatoes, *AtNHX1* gene overexpression improved K^+^ retention in cells under higher salt stress (He and He, 2023). Similarly, in transgenic tobacco, the expression of the TNHXS1-IRES-TVP1 bicistronic transcriptional unit led to an increase in the accumulation of K^+^ and a decrease in N^+^ concentration in leaf tissue (Gouiaa et al., 2012). An increase in antioxidant activities such as SOD, POD, and CAT prevents ROS accumulation and reduces cellular damage in plants (Ahmad et al., 2022a). In rice *KDML*, *RD6*, and *SKC1* genes reduced the adverse effect of ROS and increase seedling growth due to the higher antioxidant enzymes activities under salt stress (Pamuta et al., 2022). The possible results might be due to the *SKC1* genes responsible for the maintenance of ionic homeostasis, maintaining membrane integrity, and coping against salt-induced damages such as ROS detoxification (Nounjan et al., 2018; El Moukhtari et al., 2020). Hence, genes alleviated the negative effects of salt stress by improving physiological and biochemical activities of the plants. However, these gene’s roles and underlying mechanisms in various crops remain unclear under salt stress.

### Effects of salt stress on crop yield and yield-related genes

The world population is expected to increase by 34 percent in 2050, and the requirements for food production are expected to increase by 34 percent to meet the demand for cereals (FAO, 2017). This growth in cereal productivity will need to occur in a world with higher salt stress, where regular higher salt stress negatively affect plant yield (Adil et al., 2022). Hence, to improve the yield of cereals, increase the current germplasm’s yield and improve yield stability through enhanced tolerance to salt stress (Hu and Schmidhalter, 2023). It has been demonstrated that better management of land and the introduction of new genotypes through genetic engineering and breeding programs can lead to advances in yield. Recently, various approaches, traditional and state-of-the-art amelioration, have been put forward to improve plant yield (Panagea et al., 2016). Plant breeding has played a paramount role in maintaining food security, leading to increased plant productivity over the past few decades. For salt reclamation, breeding is considered one of the most efficient strategy for improving plant tolerance against salt stress (Ashraf and Munns, 2022). Despite much research documented in understanding the response of plants to salt stress, the breeding of salt-resistant genotypes remains slow, with limited progress in plants (Asif et al., 2018; Kotula et al., 2020). Compared to the slow breeding progress, to enhance the salt tolerance of barley and wheat, salt-resistant genotypes have been introduced and commercialized in various Asian countries via conventional breeding methods (Ismail and Horie, 2017). The improved rice genotypes development through this breeding method increase grain yield production in fields under salt-affected areas by 0.5 to more than 2 tons per hectare (Singh et al., 2016). Nevertheless, the development of these varieties took 5-10 years of rigorous evaluation of many breeding lines with high process costs; hence, various approaches, such as genome-based and marker-assisted breeding, are becoming more promising and attractive (Thomson et al., 2012). Different studies have shown that yield of transgenic wheat and barley genotypes with salt resistance can be developed by manipulating the expression of introducing genes or native genes (Hu and Schmidhalter, 2023). The green revolution gene, *sd1/Rht1*, significantly increased plant’s yield when it was successfully adopted in cereals (Zheng et al., 2022). In addition, the highly expressed *AtNHX1* gene in transgenic wheat lines, a gene encoding an *Arabidopsis* vacuolar Na^+^/H^+^ antiporter, showed a higher grain yield in saline field (Sharma et al., 2022). A few dwarf genes, such as *BnaMAX1s*, are involved in hormone biosynthetic activities and increased plant yield in *brassica napus* L. (Zheng et al., 2020). Similarly, rice over-dominance *ipa1-1D* and tomato *sft* genes increased plant photosynthetic activities, enhanced dry matter accumulation in sink, and increased yield (Jiao et al., 2010; Krieger et al., 2010). Nevertheless, the identified genes against salt resistance have not yet been transferred into relevant commercial genotypes nor used to generate a salt-resistant plant (Asif et al., 2018). The reason is that quantitative trait loci (QTLs) or genes have only been checked in controlled growth conditions with short periods of salt stress, which do not reflect realistic field conditions (Singh et al., 2021). Therefore, in the future, more field trials are required to measure the value of these genes and dwarf-related genes in breeding to achieve higher yield under salt stress. Moreover, clustered regularly interspaced short palindromic repeats (CRISPR-Cas9), transcription activator-like effector nucleases (TALENs), and zinc-finger nucleases (ZFNs) are techniques that easily modify genetic loci or multiple homologous genes.

## Practices of alleviating salt stress on crops

### Different technologies are used for gene modification

Genome editing technologies, which can change the target genes of the plant genome, is increasingly preferred for use in different fields, including crop breeding and plant science. Genome-editing technologies characterize crop improvement and gene function (Xia et al., 2021). The leading three technologies used in genome editing such as TALENs, ZFNs, and CRISPR/Cas9. TALENs and ZFNs are time-consuming and require a lengthy protocol to gain the specific target (Liu et al., 2021). Compared with TALENs and ZFNs, the CRISPR-Cas9 techniques are more convenient, easy to design, and vigorous (Chennakesavulu et al., 2021). CRISPER-Cas9 is a simple toolkit that is easy to design because of the involvement of only single-guided RNA (sgRNA) and the cas9 protein compared to TALENs and ZFNs (Razzaq et al., 2019). Additionally, the procedure involved in TALENs and ZFNs is complicated because they need protein engineering for their construction (Razzaq et al., 2019). Due to this obstacle, the tools of TALENs and ZFNs in plants have been limited (Jinek et al., 2012). In several plants, continuous innovation for efficient genome editing has expanded the application of CRISPR-Cas9 and is rapidly becoming a promising tool for gene modifications (Shan et al., 2013; Zhang et al., 2019). CRISPR-Cas9 (CRISPR-associated) is a prokaryotic adaptive immune system that binds and cleaves foreign nucleic acids (Brouns et al., 2008). The type II CRISPR system most frequently used is composed of two components, such as Cas9 nuclease and an artificial single guide RNA (sgRNA) (Jinek et al., 2012). CRISPR-Cas9 plays a vital role in improving plant quality and yield. Plant yield is a complicated, multigenic, and quantitative characteristic affected by various features. The CRISPR-Cas9 technique has proven to be effective in increasing plant yield. The CRISPR-Cas9 genome editing technique is only important for gene knock-in and knock-out, not for the base version (Chennakesavulu et al., 2021). The corresponding findings of (Li et al., 2017; Zhang et al., 2018) revealed that genes negatively regulate yield traits such as tiller number by *OSAAP3*, and grain size by *OsGRF*, used CRISPR-Cas9 to knock out multiple genome yield-related genes, including *Hd2*, *Hd4*, and *Hd5* (Li et al., 2017; Zhang et al., 2018). Recently, 30 genotypes of “Green Revolution miracle rice” were investigated through genome sequencing, and 57 genes controlling yield-relevant traits were knocked out via the CRIPR-Cas9 technique (Huang et al., 2018). Phenotyping results showed that many genes identified during screening were crucial to regulate yield-related traits in rice. However, more studies need further investigation to identify various genes in other crops via CRISPR-Cas9.

Abiotic stress tolerance through CRISPR-Cas9-mediated genome editing has been documented in *Arabidopsis*, wheat, rice, tomatoes, barley, and sorghum (Gobena et al., 2017; Wang et al., 2017; Sánchez-León et al., 2018; Lawrenson and Harwood, 2019; Liu et al., 2019; Tran et al., 2021). In tomatoes, the functional domains of hybrid proline-rice protein 1 (S1HyPRP1), a negative regulator of salt stress resistance, were disrupted using a CRIPR-Cas9 mediated multiple genome editing approaches (Tran et al., 2021). Further investigation showed that the precise elimination of S1HyPRP1 functional domains in tomatoes led to higher salt tolerance during all growth stages (Tran et al., 2021). The *Slmapk3* editing gene, via CRISPR-Cas9, exhibited lesser ROS, higher enzyme activities, lower membrane damage, and reduced severe plant welting under heat stress (Yu et al., 2019). Several studies have been conducted about the role of CRISPR-Cas9 in genome editing that enhanced different plant’s growth and yield, but further research is needed to investigate the performance of these modified genes via CRISPR-Cas9 techniques in different crops under salt stress.

## Selection of suitable cultivars under salt stress

### Different strategies assist in the improvement of suitable cultivars under salt stress

**Figure 4:**
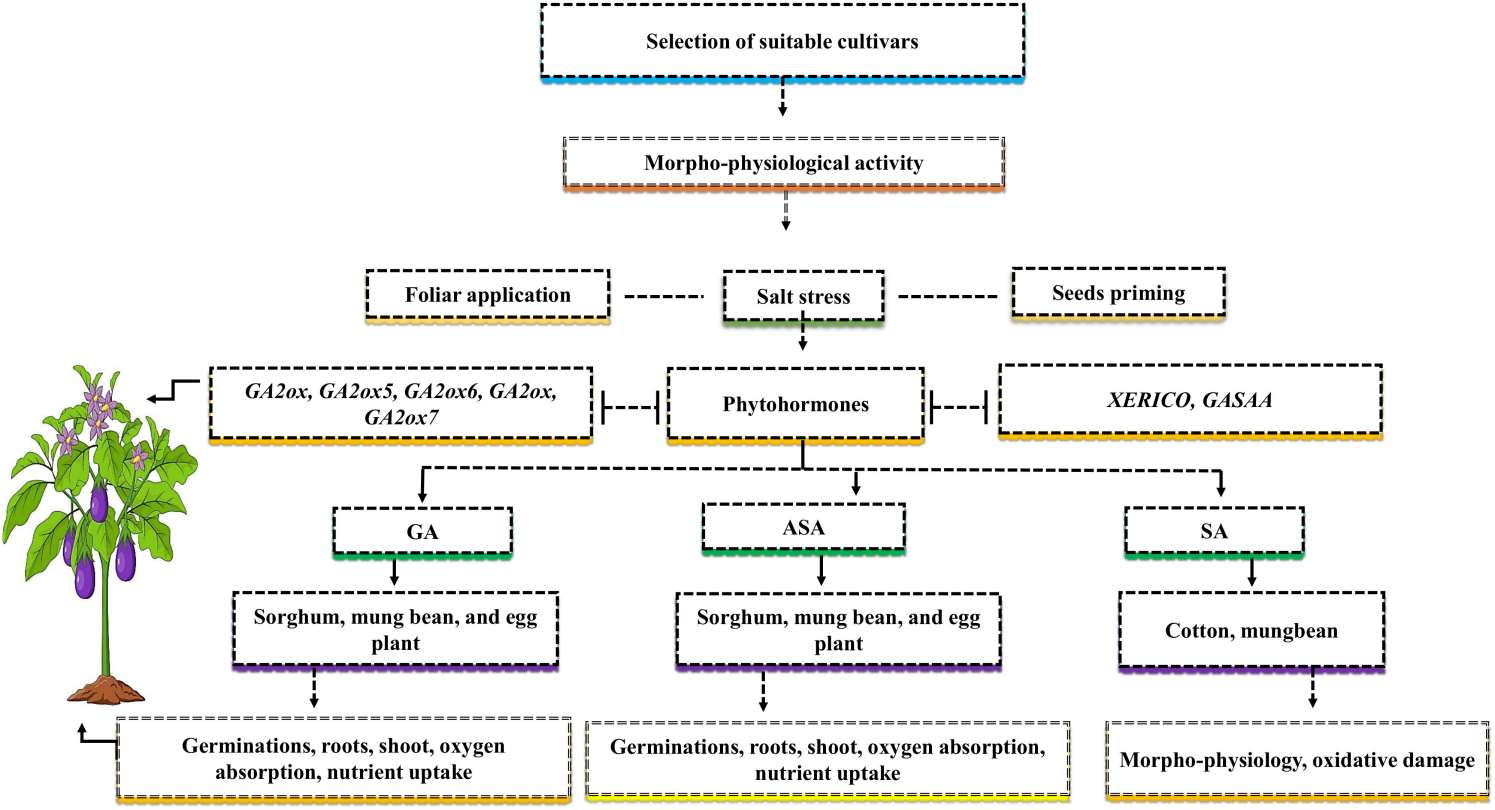
The selection of suitable cultivars can mitigate the negative effects of salt stress. For higher yield, it’s important to know the morph-physiological activity of the cultivars. The phyto-hormones such as gibberellins (GAs), ascorbate (ASA), and salicylic acid (SA) can mitigate the adverse effects of salt stress via two application methods such as foliar and seed priming. The application of GAs improves germinations, roots, shoot, oxygen absorption, and nutrient uptake in sorghum, mung bean, and eggplant, ASA improves germinations, roots, shoot, oxygen absorption, nutrient uptake in sorghum, mung bean, and eggplant, and SA improves morpho-physiology activities, and oxidative damage in cotton and mung bean. The genes such as *GA2ox, GA2ox5, GA2ox6, GA2ox, GA2ox7, XERICO* and *GASAA* are involved in endogenous phyto-hormones and increase plant yield.

Salt resistance relies on the selection of suitable genotypes and their families (**Figure 4**). Investigating the comparison or examining the physiological mechanism of salt stress in some cultivars that belong to the same family can provide more knowledge of the discriminative growth pattern of plant and salt resistance levels in both cultivars (Shahzad et al., 2021). Hence, due to the lack of examination of physiological mechanisms concerning salt stress in genotypes, it is critical to determine the negative impact of salt stress on crop growth and yield. Adapting cultivars to salt stress involves complex biochemical, physiological, and molecular mechanisms, which are still in an early phase (Denaxa et al., 2022). To reduce the negative effects of salt stress on crop growth and development, various strategies, like seed priming and foliar application, should be used to understand plant morpho-physiological and biochemical activities (**Figure 4**). Seed priming is when various crop seeds are soaked with one or more growth regulators at an appropriate level before they are sown.

In contrast, the foliar application is the treatment of one or more growth regulators applied at an appropriate level in a liquid form directly to the leaves. Previous studies demonstrated that NaCl reduced germination percentage in three beans and two sorghum cultivars (Hasanuzzaman et al., 2020). The reduction in germination percentage might be caused by increased NaCl osmotic pressure, which slows down the water imbibition, germination and metabolism processes of the seeds of sorghum and beans (Xie et al., 2019). The application of ASA enhanced the germination percentage of sorghum and eggplant under the higher level of NaCl. The treatment reduced the adverse effects of salt stress, improved oxygen absorption, and improved the transportation of nutrients from cotyledon to embryos (Irfan et al., 2021). Plant roots and shoots are considered essential parameters for salt stress because roots come into contact with the soil and absorb water from it and transfer it to the shoots. For instance, the reports of Xie et al. (2019) on various plants and Hussien Ibrahim et al. (2020) on sorghum showed that tissue alteration under salt stress caused a significant decrease in the seedling growth characteristics (Xie et al., 2019; Hussien Ibrahim et al., 2020). The application of exogenous ASA increased sorghum seedling growth characteristic while mitigating the negative effects of salt stress (Mohammed Ibrahim Elsiddig et al., 2022). Similar results were supported by Mittal et al. (2018) in *Brassica rapa* L., who demonstrated that seed treatment with ASA before sowing has dramatically improved seedling growth characteristics and protects the plants roots and shoots form altering (Mittal et al., 2018). The improved seedling growth characteristic might be due to the ascorbic acid antioxidant action and or increased cell enlargement within the apical meristem of seedlings (Wang et al., 2019).

Moreover, seed priming with suitable amount of GA can protect against seed deterioration and mitigate the adverse effects of salt stress, such as ion toxicity, osmotic stress, and an imbalance of nutrients uptake (Ahmad et al., 2022b). Seeds soaked with GA application facilitate germination and increased seedling length in rice and sorghum, as GA stimulated cell division and cell elongation (Saudi, 2017; Shihab and Hamza, 2020). Similarly, SA applications can enhance plant tolerance to salt stress in different crops (Khan et al., 2015). Such as, the reasonable concentration of SA in stressed plants via seeds soaking before sowing and after germination via spraying or adding to the nutrient solution can improve the morpho-physiological activities of cotton seedlings under salt stress (Ahmad et al., 2022a). SA improved mung beans growth characteristics and photosynthetic activities, and at the same time, oxidative damage from salt stress was reduced (Ahanger et al., 2019). The seed priming and foliar application of ASA, GA, and SA play an important role in enhancing salt tolerance mechanism and promoting the germination of *Medicago sativa* L. *Brassica juncea* L. and cotton (Ahmad et al., 2022b). Further studies are required to close the knowledge gap regarding the application of ASA, GA, and SA to other growth attributes, such as the physiological and biochemical characteristics of various plants via other molecular techniques.

Additionally, to improve plant growth and yield with the help of the selection of suitable cultivars, it is crucial to understand the genes in plants that are responsive to phyto-hormones. In response to salt stress, the genes *GA2ox7* in cotton, *XERICO* and *GASAA* in *Arabidopsis thaliana*, *GA2ox, GA2ox5*, and *GA2ox6* in rice, and *GA2ox* in potatoes were upregulated by GAs (Ahmad et al., 2022b). These genes support the growth and development of plants and are involved in a number of biological processes. The *GA2ox7* genes enhance the content of abscisic acid (ABA) and indole acetic acid (IAA) in cotton (Ahmad et al., 2022b). The expression of *GA2ox6* genes in rice transgenic plants increased the grain yield by 10-30% during abiotic stresses (Lo et al., 2017). These genes are involved in plant endogenous hormones, but their signaling and transduction pathways in different species under salt stress are still not being clearly understood.

## Conclusions and future recommendations

The impact of salt stress on plant growth is considered a significant threat to agricultural productivity. Salt stress mainly reduces the plant’s growth through physiology and the imbalance of ion homeostasis, which can alter gene expression. Therefore, to enhance the yield of plants under salt stress conditions, it is crucial to understand the integrative approaches. Salt stress is a highly complicated process, and coping with the negative effects of this stress is still poorly understood.

The application of HA with K^+^ improves morpho-physiological activities and soil properties. However, its performance with N, P, and other molecules such as FA, are far from clear.

The current study investigated that phyto-hormones such as ASA, SA, and GA improve the growth attributes in several plants. However, the detailed mechanism of these phyto-hormones in physiological and biochemical activities in different plants under salt stress is still in an early phase.

Stomatal conductance is paramount in net photosynthesis activities in several crops, such as wheat, barley, and sorghum. However, elucidating the detailed mechanism of stomatal conductance under salt stress in various crops is still unknown.

Gene families such as *NRT* and *NPF* uptake nitrate and translocate it to other parts of the plants. However, the performance of these genes family, especially the *NPF6.3, NRT1.1/CHL1* under salt stress in different crops is still unknown.

It has been confirmed that various genes, including *NHX1, SOS1, TaNHX, AtNHX1, KDML, RD6*, and *SKC1*, maintain ion homeostasis and membrane integrity to cope with salt-induced damage in different plants. However, these genes’ performance and underlying mechanisms in several crops remain unknown under salt stress.

Different genes, such as *sd1/Rht1, AtNHX1, BnaMAX1s, ipal-1D*, and *sft*, improved the growth and yield of various plants. However, the identification of these genes against salt tolerance has not yet been transferred into the relevant commercial genotypes or used to generate salt-resistant plants.

CRISPER-Cas9 successfully knocks out various genes such as *OSAAP3*, *OsGRF4, OsGS3, TaGW2*, *TaGASR7, Hd2, HD4*, and *Hd5*, which negatively regulate the yield traits of rice. In contrast, the genes modified by CRISPR-Cas9-modified genes *Slmapk3* enhanced enzyme activities, reduced plant wilting, and increased plant yield under heat stress. However, further studies require to investigated to knock in or knock out different genes via CRISPR-Cas9 under salt stress.

## Author contributions

IA: Visualized the idea, participated in the writing (review and editing), and arranged the contents and draft of the original manuscript. GSZ and GLZ: Acquired funding and contributed to the reviewing and editing of the manuscript. JL, YZ and MY: Assisted in the collection of literatures. ES and MS: Polished the manuscript and eliminated grammatical errors. All authors contributed to the article and approved the submitted version.
